# Supplementary material for: PCP and Wnt pathway components act in parallel during zebrafish mechanosensory hair cell orientation
Source: Nat Commun. 2019 Sep 5;10:3993. doi: 10.1038/s41467-019-12005-y (PMC6728366; doi:10.1038/s41467-019-12005-y)
Supplement: Supplementary file 1 — Supplementary Information [file 41467_2019_12005_MOESM1_ESM.pdf]

Supplementary Information

‘PCP and Wnt pathway components act in parallel during zebrafish mechanosensory hair cell orientation'

Navajas Acedo, *et al.*

Supplementary Figure 1

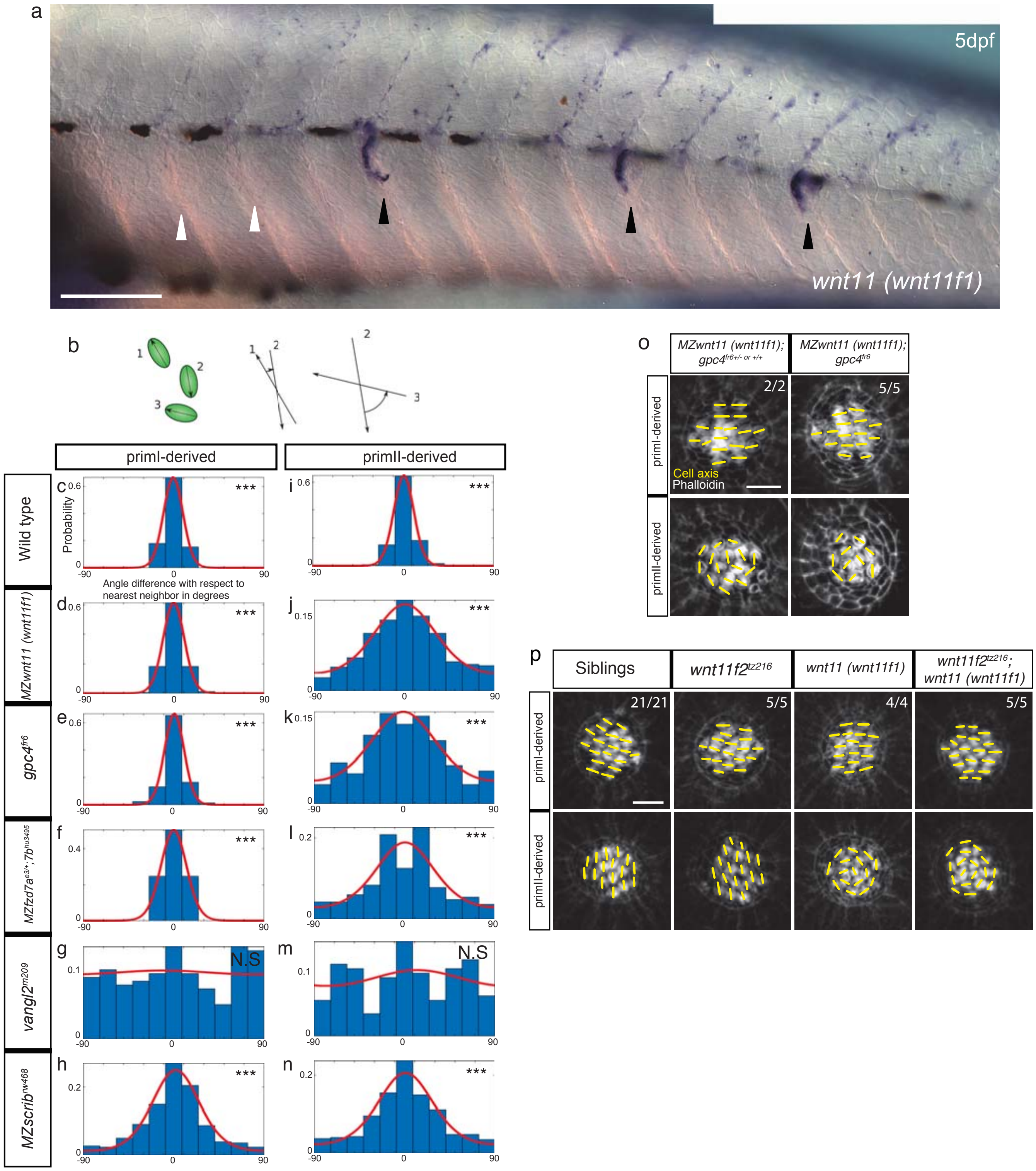

Supplementary Figure 1: *wnt11 (wnt11f1)* is expressed in the lateral line, interacts with *gpc4* and does not act redundantly with *wnt11f2* in setting up hair cell orientation.

a. PrimI-derived neuromasts are labeled with black arrowheads. White arrowheads indicate PrimII-derived neuromasts that are outlined by using a dotted line. PrimI-derived neuromasts show *wnt11 (wnt11f1)* mRNA expression on the anterior side but no signal is present in primII-derived neuromasts (see also Fig. 1d). **b-n**. Analysis of hair cell orientation with respect to the nearest neighbor. **b**. Diagram showing how the angle between two neighboring hair cells was calculated based on their relative positions to each other. For more details, see Methods. **c-n**. Histograms of angular data and fitted distribution (red) for each of the conditions analyzed. **c-h**. Binned angle distribution in primI-derived neuromasts. The von Mises distribution is shown in red to display the distribution of the data. **c**. Distribution in primI-derived neuromasts of wild type fish. Note the tight degree of alignment between neighbors (Uniform distribution p-value=7.75 *x* 10^-107). **d-f**. Distribution in primI-derived neuromasts of Wnt pathway mutants. They all show a high degree of alignment (Uniform distribution p-value in c= 1.21 *x* 10^-277, d= 1.97 *x* 10^-184 and e=5.46 *x* 10^-44). **g**. Angle distribution in primI-derived neuromasts of *vangl2* mutants. Neighbor hair cells do not show angle coordination (Uniform distribution p-value= 0.212). **h**. Angle distribution in primI-derived neuromasts of *MZscrib* mutants. Hair cells show a high degree of coordination between neighbors (Uniform distribution p-value= 7.11 *x* 10^-86), but the distribution is more dispersed than the one shown by wild type fish. **i-n**. Binned angle distribution in primII-derived neuromasts. **i**. Angle distribution in primII-derived neuromasts of wild type fish. Note the high degree of alignment between neighbor hair cells (Uniform distribution p-value= 2.08 *x* 10^-106). **j-l**. Angle distribution in primII-derived neuromasts of Wnt pathway mutants. In all three conditions, hair cells show significant degree of coordination between neighbors (Uniform distribution p-value in i= 3.09 *x* 10^-18, j= 1.97 *x* 10^-07, k= 7.03 *x* 10^-14). **m**. Angle distribution in primII-derived neuromasts of *vangl2* mutants. Neighbor hair cells do not show coordination in their alignment (Uniform distribution p-value= 0.059). **n**. Angle distribution in primII-derived neuromasts of *MZscrib* mutants. Hair cells show a high degree of coordination between neighbors (Uniform distribution p-value= 1.09 *x* 10^-33), but the distribution is more dispersed than the one shown by wild type fish. **o**. Hair cell orientation is disrupted only in primII-derived neuromasts of 5dpf *MZwnt11 (wnt11f1)* homozygous siblings and *MZwnt11 (wnt11f1);gpc4* double homozygous fish. Double *MZwnt11 (wnt11f1);gpc4* mutants do not show a hair cell phenotype in primI neuromasts. **p**. Hair cell orientation in 5dpf *wnt11f2* homozygous, *wnt11 (wnt11f1)* homozygous and double *wnt11f2;wnt11 (wnt11f1)* homozygous fish. Only a mutation in *wnt11 (wnt11f1)* disrupts hair cell orientation in primII-derived neuromasts. Thus, *wnt11 (wnt11f1)* and *wnt11f2* do not act redundantly during the establishment of hair cell orientation. The cell axis defined by the position of the kinocilium is labeled in yellow. Scale bar in **a** equals 50μm, scale bar in **o** and **p** equals 5μm.

Supplementary Figure 2

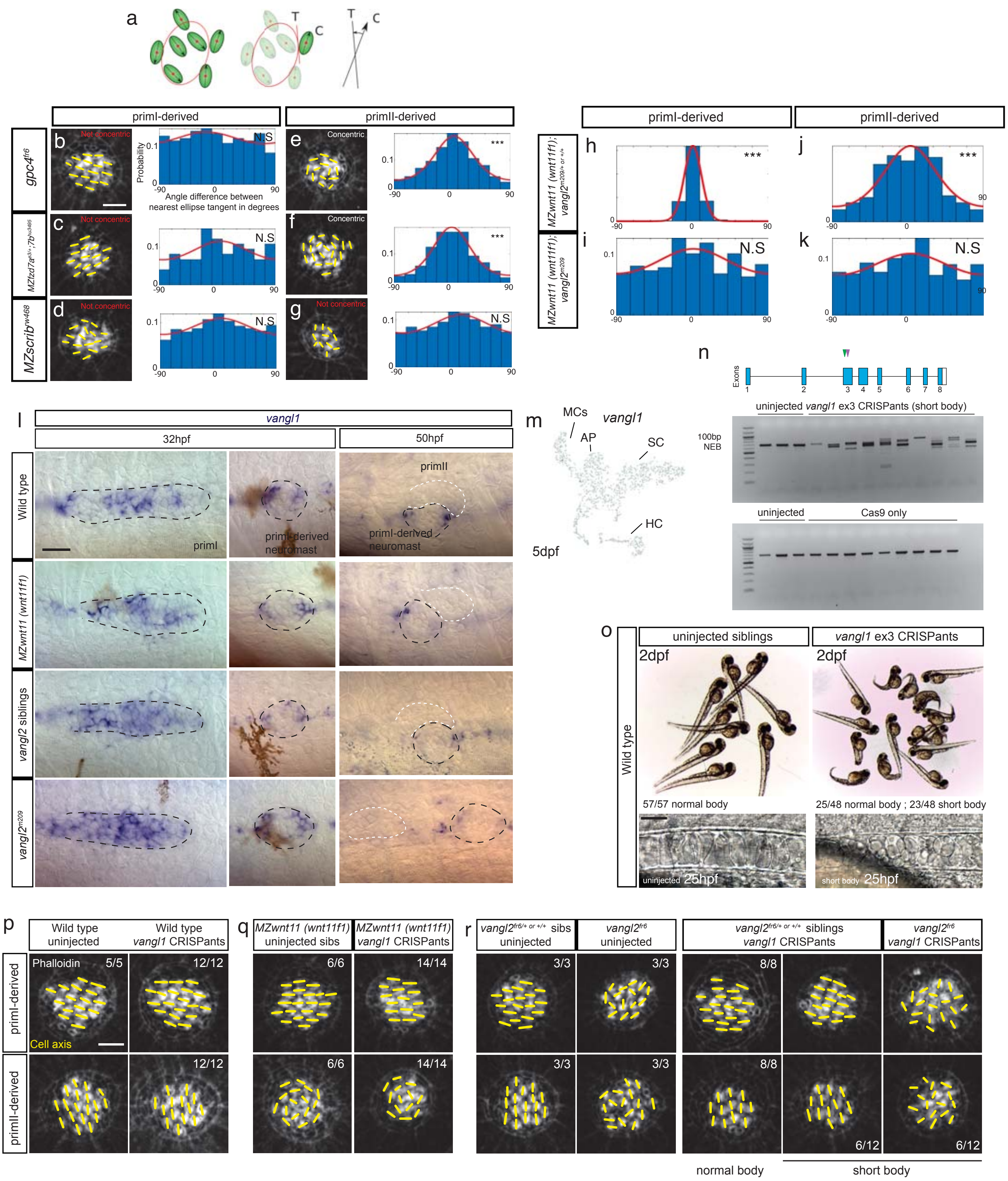

Supplementary Figure 2: Wnt and PCP pathway mutants show different hair cell phenotypes, but not due to redundancy with *vangl1.*

a. Diagram showing how the analysis of angle difference between a given hair cell's orientation and the nearest fitting ellipse (concentricity) in each of the conditions was calculated. The angle between a cell's axis (C) with respect to the tangent (T) of the nearest fitted ellipse is calculated for each cell. For more details, see Materials and Methods. **b-d**. Phalloidin images showing the cell polarity axis (yellow lines) in primI-derived neuromasts of *gpc4* (**b**), *MZfzd7a*/7b (**c**) and *MZscrib* mutants (**d**). Based on the distribution of angles with respect to the nearest ellipse tangent (concentricity) none of the mutants shows significant concentricity (Uniform distribution p-values in b= 0.95, c= 0.666, d=0.165; *gpc4* n=222 hair cells, *MZfzd7a*/7b n=74, *MZscrib* n=535). Additionally, the von Mises distribution is shown in red for visual display of the data distribution. **e-g**. Phalloidin images showing the cell polarity axis (yellow lines) in primII-derived neuromasts of *gpc4* (**e**), *MZfzd7a*/7b (**f**) and *MZscrib* (**g**) mutants. Only the Wnt pathway mutants show significant concentricity (Uniform distribution p-values in e= 2.03 *x* 10^-11, f= 2.05 *x* 10^-12, g=0.488; *gpc4* n=214 hair cells, *MZfzd7a*/7b n=182, *MZscrib* n=392). Yellow lines in b-g indicate the hair cell polarity axis, determined by the position of the kinocilium. Not Concentric vs Concentric labels were based on statistical significance. **h-k**. Analysis of hair cell orientation with respect to the nearest neighbor. **h-i**. Angle distribution in primI-derived neuromasts of single *MZwnt11 (wnt11f1)* siblings (**h**) and *MZwnt11 (wnt11f1)*;*vangl2* mutants (**i**). The high degree of alignment in h (Uniform distribution p-value= 1.82 *x* 10^-223) is lost in i (Uniform distribution p-value= 0.0395). **j-k**. Angle distribution in primII-derived neuromasts of single *MZwnt11 (wnt11f1)* siblings (**j**) and double *MZwnt11 (wnt11f1)*;*vangl2* mutants (**k**). Note how the high degree of alignment in j (Uniform distribution p-value= 1.29 *x* 10^-14) is lost in k (Uniform distribution p-value= 0.069). **l**. *vangl1* in situ in wild type, *MZwnt11 (wnt11f1)* mutants and *vangl2* siblings and mutants. No evident changes in expression were observed. **m**. scRNAseq t-SNE plot for *vangl1* expression in a 5dpf wild type neuromast, where its expression is low in mantle cells (MCs). **n**. Agarose gels showing the efficiency of CRISPR targeting of exon 3 in *vangl1* in CRISPants (upper part) and Cas9-only injections (lower part). **o**. Brightfield images of 2dpf wild type uninjected siblings (left) and *vangl1* CRISPants (right), which show shortened, curly tails. Bottom panels: DIC images of 25 hpf live embryos showing the morphology of the notochord cells that are stacked in uninjected fish and are rounded in *vangl1* CRISPants, a characteristic of PCP defects. **p-r**. Phalloidin staining of the hair cell orientation in uninjected and *vangl1* CRISPR injected wild type (**p**), *MZwnt11 (wnt11f1)* (**q**) and *vangl2* mutants (**r**). *vangl1* targeting by CRISPR does not affect hair cell orientation in wild type fish or modifies the phenotype characteristic of *MZwnt11 (wnt11f1)* and *vangl2* mutants. Scale bar in **b** and **p** equals 5µm; **l** and **o** equals 20µm.

Supplementary Figure 3

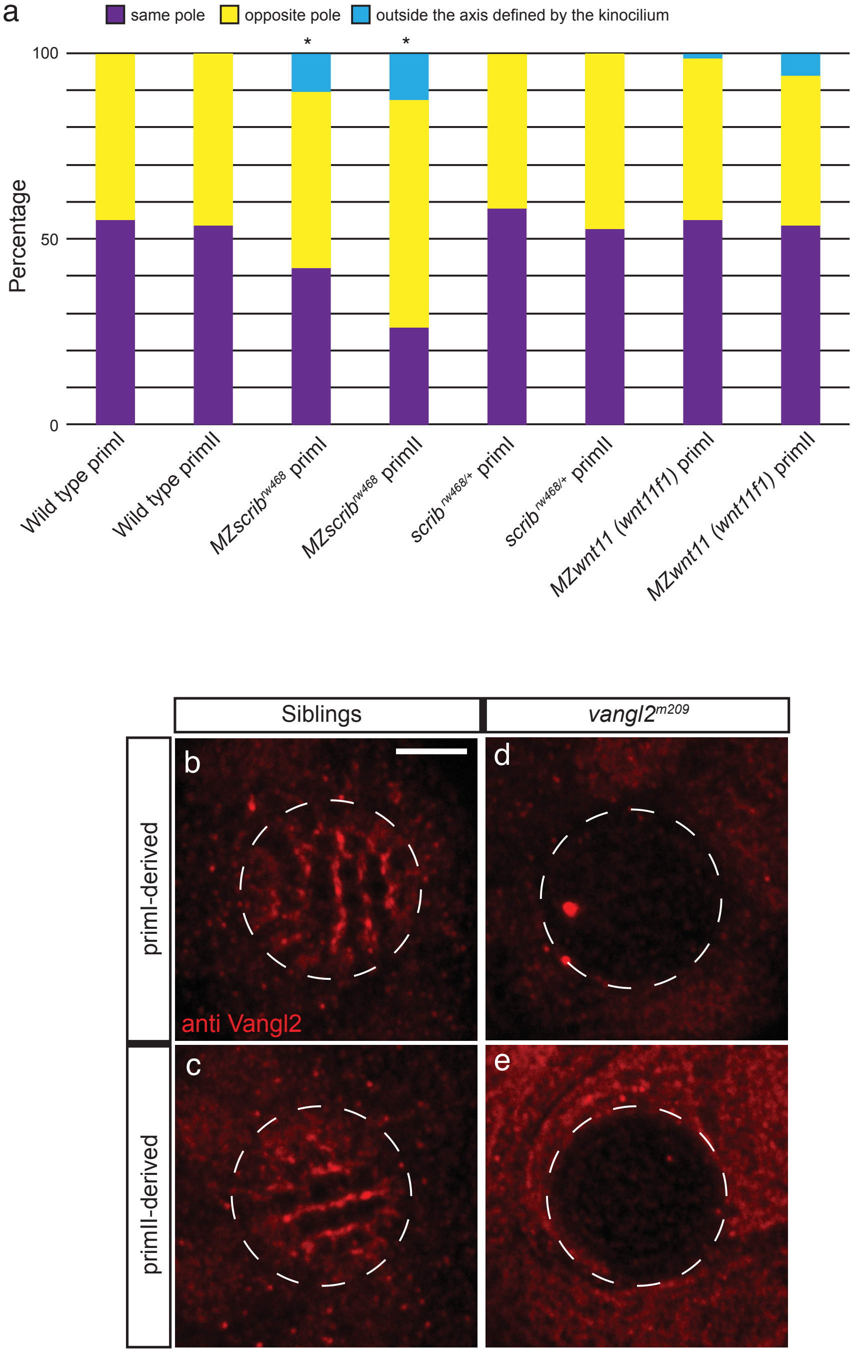

Supplementary Figure 3: Vangl2 enrichment analysis in Wild type and the different Wnt and PCP pathway mutants.

a. In all the hair cells that show asymmetric Vangl2 in Figure 3i (green bar), we measured the pole of enrichment with respect to the position of the kinocilium. The results are categorized into three possibilities: asymmetric in the same pole as the kinocilium, asymmetric in the opposite pole than the kinocilium, and asymmetric but outside of the axis defined by the kinocilium. We observe differences between *MZscrib* and their siblings (Fisher's exact test p-val WT primI vs *MZscrib* primI= 0.09724; p-val *scrib* siblings primI vs *MZscrib* primI= 0.08285; p-val WT primII vs *MZscrib* primII= 0.02568; p-val *scrib* siblings primII vs *MZscrib* primII= 0.03557). Wild type and *MZwnt11 (wnt11f1)* mutants do not show differences (p-val WT primI vs *MZwnt11 (wnt11f1)* primI= 1; p-val WT primII vs *MZwnt11 (wnt11f1)* primII= 0.439). **b-e**. Vangl2 antibody staining on *vangl2* siblings primI (**b**) and primII (**c**). Vangl2 antibody staining is absent in primI (d) and primII (**e**) of *vangl2* mutants. * denotes statistical significance. Scale bar equals 5μm.

Supplementary Figure 4

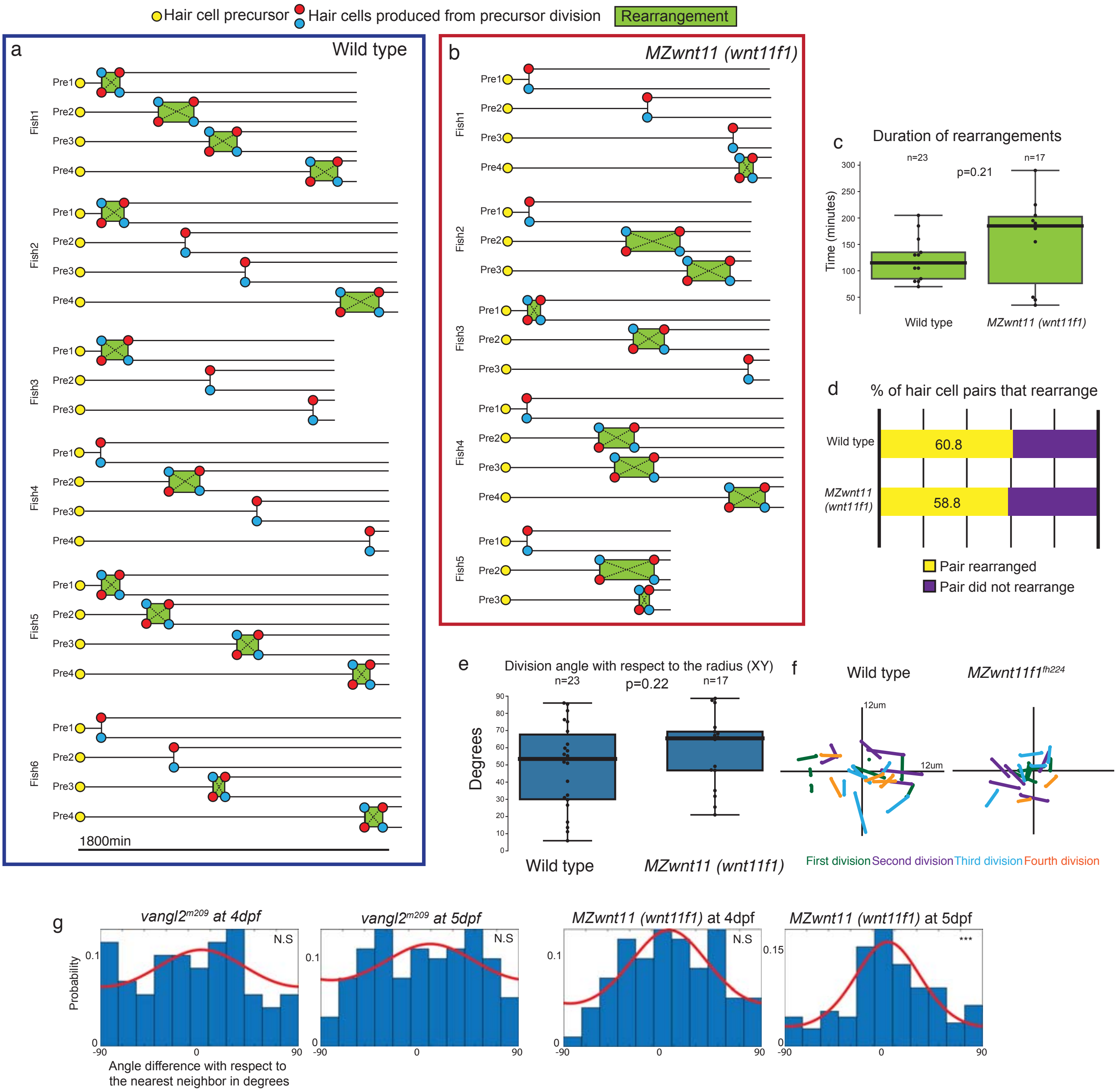

Supplementary Figure 4: Comparison between the cell behaviors and hair cell phenotypes in the Wnt and PCP pathway mutants during development.

a-f. Analyses of cell behaviors of hair cell precursors during development show no differences between Wild type and *MZwnt11 (wnt11f1)* mutants. **a-b**. Visual representation of the individual time lapses of hair cell formation in primII-derived neuromasts of Wild type (**a**) and *MZwnt11 (wnt11f1)* mutants (**b**). Each hair cell precursor (yellow) divides into two hair cells (red and blue) and either rearrange or not. **c**. Duration of the rearrangement that the hair cell progenitors undergo after division in wild type and *MZwnt11 (wnt11f1)* mutant embryos. **d**. Percentage of hair cell progenitors that undergo rearrangements after division in wild type and *MZwnt11 (wnt11f1)* mutant fish. **e**. Division angle with respect to the radius of each division event in hair cell progenitors of wild type and MZ*wnt11 (wnt11f1)* mutant fish, and (**f**) the visual representation of each division event with respect to the XY axis. **g**. Histograms of angular data and von Mises fitted distribution (red) for the quantification of the alignment of each hair cell with respect to their closest neighbor during the development of primII-derived neuromasts in *vangl2* and *MZwnt11 (wnt11f1)* mutant fish at 4 and 5dpf. Note how none of the conditions, except for *MZwnt11 (wnt11f1)* at 5dpf show coordination of the alignment of their hair cells with respect to their neighbor (Uniform distribution p-values for *vangl2* at 4dpf=0.733, *vangl2* at 5dpf=0.512, *MZwnt11 (wnt11f1)* at 4dpf=0.066, *MZwnt11 (wnt11f1)* at 5dpf=1.46 *x* 10^-04). N.S = Not significant. *** p-value < 0.01.

Supplementary Figure 5

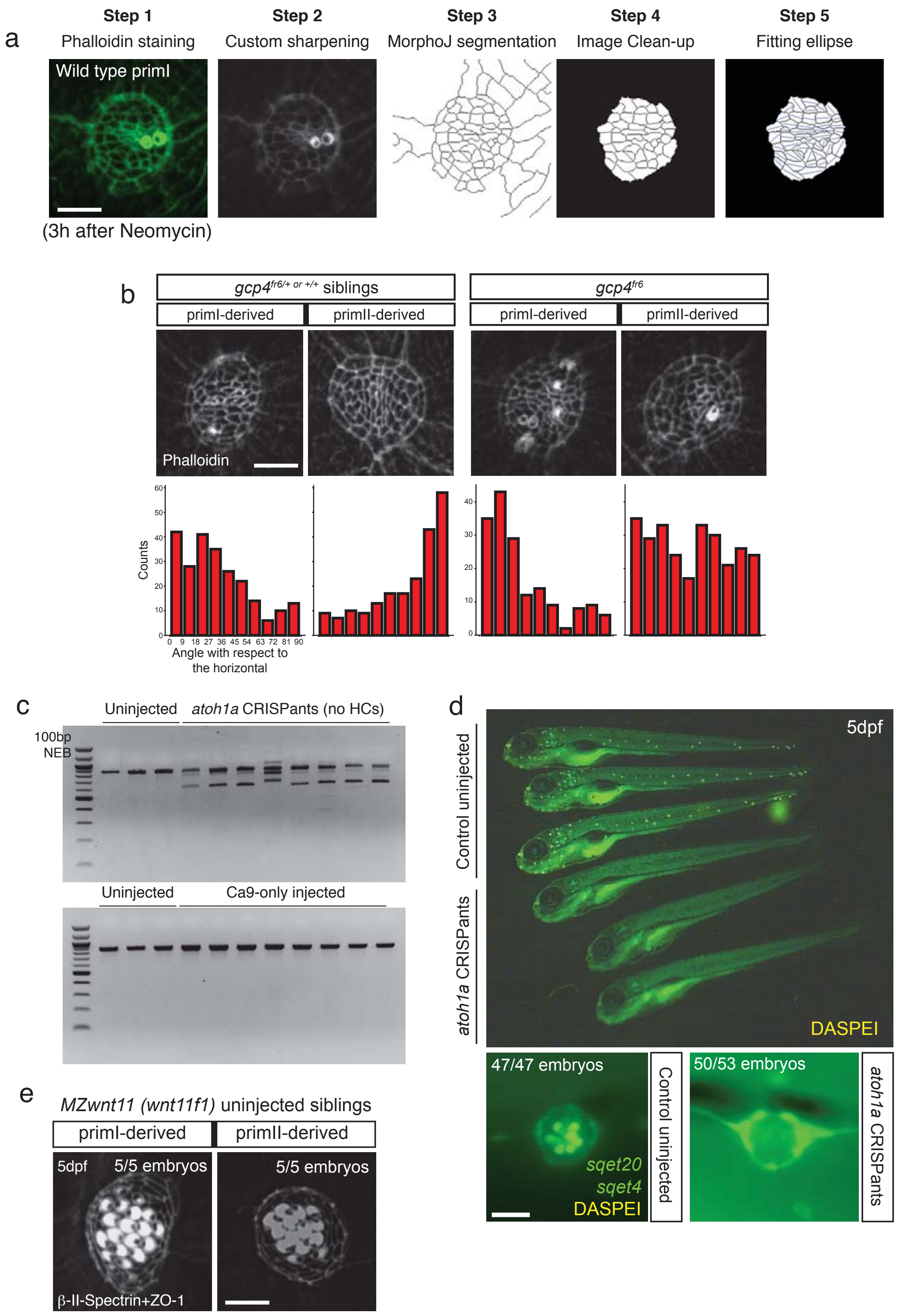

Supplementary Figure 5: Pipeline for segmentation and analysis of support cell orientation, and description of the *atoh1a* CRISPR experiments.

a. Pipeline for the segmentation of Phalloidin stainings of neuromasts 3 hours after hair cell ablation. Each Phalloidin image is sharpened and segmented. After segmentation, only the support cells are kept and used for fitting the Ellipse that determines the cell's long axis. For further information, see Materials and Methods. **b**. Phalloidin staining of 5 dpf *gpc4* sibling or mutants after removal of hair cells and quantification of support cell orientation with respect to the horizontal, showing support cell disorganization only in primII-derived neuromasts of *gpc4* mutants (Uniform distribution p-val *gpc4* siblings primI= 1.43 *x* 10^-12, *gpc4* siblings primII= 1.30 *x* 10^-14; *gpc4* mutants primI= 1.84 *x* 10^-14, *gpc4* mutants primII= 0.80). **c**. Agarose gels showing the efficiency of CRISPR targeting the single exon of *atoh1a* in CRISPants (upper part) and Cas9-only injections (lower part). **d**. DASPEI staining of uninjected and *atoh1a* CRISPR injected fish, showing that *atoh1a* CRISPants do not possess hair cells. Below are shown higher magnification pictures of the neuromasts, where *sqet20* labels the mantle cells and *sqet4* and DASPEI label the hair cells. **e**. Double β-II-Spectrin and ZO-1 labeling of the uninjected *MZwnt11 (wnt11f1)* siblings for Figure 5q, r. Scale bar in **a**, **b** and **e** equals 5 μm, **d** equals 20μm.

Supplementary Figure 6

a

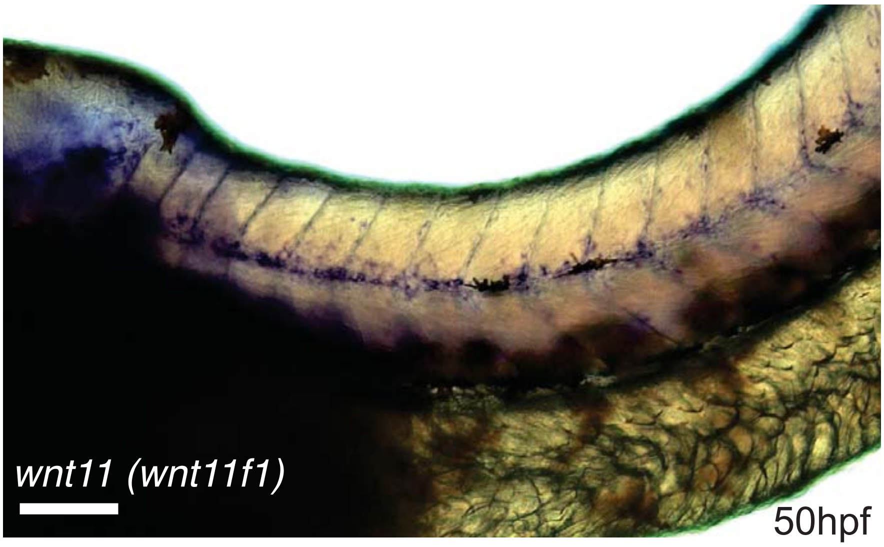

b

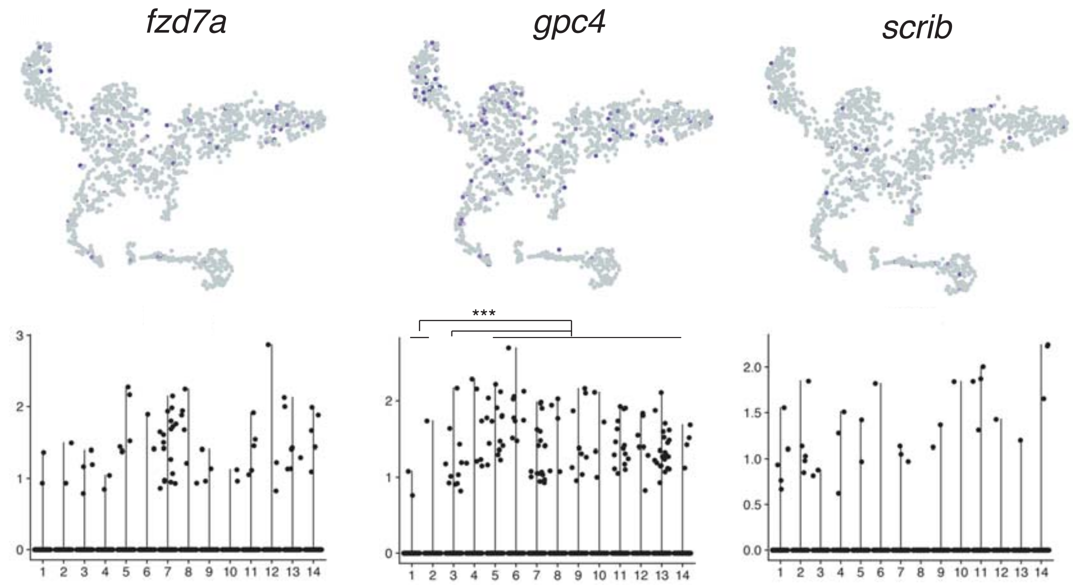

c

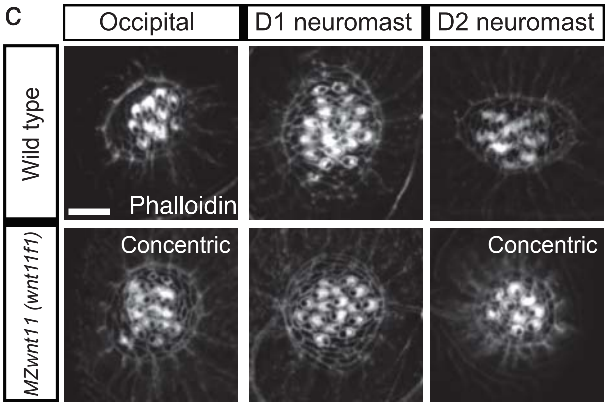

Supplementary Figure 6: Expression of the different components of the Wnt and PCP pathways, and hair cell orientation in the Occipital and Dorsal lateral lines.

a. Low magnification picture of *wnt11 (wnt11f1)* mRNA expression in the trunk of a 50hpf Wild type fish. *wnt11 (wnt11f1)* is expressed along the myoseptum. **b**. t-SNE plots and violin plots showing expression of *fzd7a*, *gpc4* and *scrib* in a 5dpf homeostatic neuromast. The Wilcox p-value for *gpc4* equals 6.4 *x* 10^-4 in support cells and mantle cells versus hair cells, while in *fzd7a* and *scrib* the expression pattern was too sparse to generate meaningful statistics. **c**. Hair cell orientation of neuromasts in the Occipital and Dorsal lateral lines of Wild type and *MZwnt11 (wnt11f1)* mutants. Neuromasts that show a hair cell phenotype in *MZwnt11 (wnt11f1)* mutants are labeled as concentric. D1=Dorsal 1. D2= Dorsal 2 (belonging to the dorsal lateral line). Scale bar in **a** equals 50μm; **c** equals 5μm.

Supplementary Figure 7

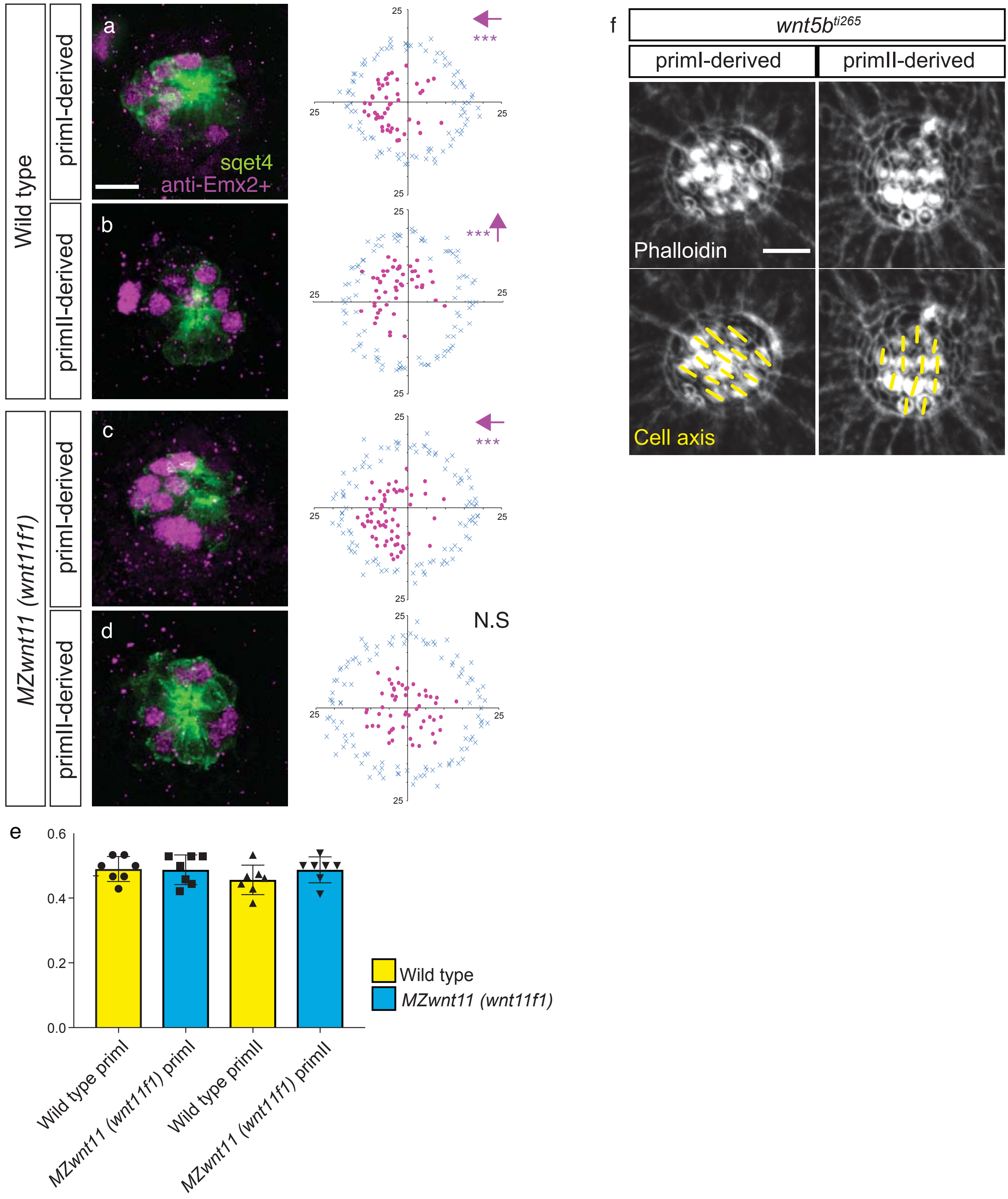

Supplementary Figure 7: Emx2+ expression analysis and hair cell orientation in *wnt5b* mutants.

a-e. Distribution, but not ratio of expression, of Emx2+ hair cells is affected in primII-derived neuromasts of *MZwnt11 (wnt11f1)* mutants. Antibody staining for the transcription factor Emx2 in 5dpf Wild type (**a-b**) and *MZwnt11 (MZwnt11f1)* mutants (**c-d**). In Wild type, Emx2+ hair cells are biased towards the anterior pole of the primI-derived neuromasts (a, Binomial test p=val 7.6 *x* 10^-06) and towards the dorsal pole in primII-derived neuromasts (b, p-val=7 *x* 10^-04). In *MZwnt11 (wnt11f1)* mutants, the distribution is still biased in primI-derived neuromasts (c, p-val=8.5 *x* 10^-10) but it is disrupted in primII-derived neuromasts (d, p-val=0.2). Additionally, in both wild type and *MZwnt11 (wnt11f1)* mutants, approximately half of the hair cells express Emx2 (e). **f**. Phalloidin staining of primI and primII-derived neuromast in a 5dpf $wnt5b^{ti265}$ mutant fish, showing no hair cell orientation defects. The arrows in **a-c** indicate the direction of bias in each case. *** denotes statistical significance for biased location. N.S= not significant. Error bars in **e** represent Standard Deviation. Scale bar in **a** equals 10μm, **f** equals 5μm.
