# Supplementary material for: PCP and Wnt pathway components act in parallel during zebrafish mechanosensory hair cell orientation
Source: Nat Commun. 2019 Sep 5;10:3993. doi: 10.1038/s41467-019-12005-y (PMC6728366; doi:10.1038/s41467-019-12005-y)
Supplement: Supplementary file 3 — Description of Additional Supplementary Files [file 41467_2019_12005_MOESM3_ESM.pdf]

Title: Supplementary Movie 1: Time lapse analysis of hair cell formation during primII migration of a ~55 hpf Wild type *Tg(cxcr4b:H2A-EGFP)* fish.

Description: The hair cell progenitor (labeled in yellow) will divide and produce two hair cells (labeled in purple and cyan). Note how the purple cell is in the dorsal pole after division and reverses position with the cyan cell. Frame rate 1/5min.

Title: Supplementary Movie 2: Time lapse analysis of hair cell orientation of a 3dpf Wild type *Tg(myo6b:β-Actin-GFP)* transgenic fish.

Description: The top down view shows the orientation of the actin-rich cuticular plates throughout the duration of the time lapse. Frame rate 1/30min.

Title: Supplementary Movie 3: Time lapse analysis of hair cell orientation of a 3dpf *MZwnt11 (wnt11f1)* mutant *Tg(myo6b:β-actin-GFP)* transgenic fish.

Description: Note how the orientations of the actin-rich cuticular plates of neighboring hair cells change over time. Frame rate 1/30min.

Title: Supplementary Movie 4: Time lapse analysis of hair cell orientation of a 3dpf *vangl2* mutant *Tg(myo6b:β-Actin-GFP)* transgenic fish.

Description: Note how the orientations of the actin-rich cuticular plates of neighboring hair cells change over time. Frame rate 1/30min.

Title: Supplementary Movie 5: Time lapse analysis of the formation of primII, primD, Occipital prim and D1 neuromast in a ~24hpf wild type *Tg(cldnb:lyn-GFP)* transgenic fish.

Description: The ear is on the left, and primI will migrate posteriorly (right) out of the field of view at the beginning of the video. The D0 placode is labeled by an arrow, and each subsequently forming primordium is labeled using an arrowhead. The D1 neuromast is labeled using an asterisk. Frame rate 1/5min.
