# Supplementary material for: PCP and Wnt pathway components act in parallel during zebrafish mechanosensory hair cell orientation
Source: Nat Commun. 2019 Sep 5;10:3993. doi: 10.1038/s41467-019-12005-y (PMC6728366; doi:10.1038/s41467-019-12005-y)
Supplement: Supplementary file 9 — Reporting Summary [file 41467_2019_12005_MOESM9_ESM.pdf]

nature research

Corresponding author(s): Tatjana Piotrowski, PhD

Last updated by author(s): May 20, 2019

Reporting Summary

Nature Research wishes to improve the reproducibility of the work that we publish. This form provides structure for consistency and transparency in reporting. For further information on Nature Research policies, see Authors & Referees and the Editorial Policy Checklist.

Statistics

For all statistical analyses, confirm that the following items are present in the figure legend, table legend, main text, or Methods section.

n/a	Confirmed	
☐	☒	The exact sample size (*n*) for each experimental group/condition, given as a discrete number and unit of measurement
☒	☐	A statement on whether measurements were taken from distinct samples or whether the same sample was measured repeatedly
☒	☐	The statistical test(s) used AND whether they are one- or two-sided<br>*Only common tests should be described solely by name; describe more complex techniques in the Methods section.*
☒	☐	A description of all covariates tested
☒	☐	A description of any assumptions or corrections, such as tests of normality and adjustment for multiple comparisons
☐	☒	A full description of the statistical parameters including central tendency (e.g. means) or other basic estimates (e.g. regression coefficient) AND variation (e.g. standard deviation) or associated estimates of uncertainty (e.g. confidence intervals)
☐	☒	For null hypothesis testing, the test statistic (e.g. *F*, *t*, *r*) with confidence intervals, effect sizes, degrees of freedom and *P* value noted<br>*Give P values as exact values whenever suitable.*
☐	☒	For Bayesian analysis, information on the choice of priors and Markov chain Monte Carlo settings
☒	☐	For hierarchical and complex designs, identification of the appropriate level for tests and full reporting of outcomes
☒	☐	Estimates of effect sizes (e.g. Cohen's *d*, Pearson's *r*), indicating how they were calculated

Our web collection on statistics for biologists contains articles on many of the points above.

Software and code

Policy information about availability of computer code

Data collection	Raw data for our previously published single cell RNA-seq data set (Lush et al. 2019) can be found at the Gene Expression Omnibus (GEO) data base (accession no. GSE123241).
Data analysis	Hair Cell orientation analysis (alignment between neighbors and concentricity): https://github.com/ThomasEWoolley/Cell_alignment<br>Support Cell orientation analysis: https://github.com/richard-alexander/neuromast_cell_orientation<br><br>Imaris x64 9.2.1 was used to calculate hair cell progenitor behaviors during development.<br><br>The web application for the single cell RNA-seq can be found at https://piotrowskilab.shinyapps.io/neuromast_homeostasis_scrnaseq_2018/. The source code for this application can be found at https://github.com/Piotrowski-Lab/Shiny-Apps/tree/master/neuromast_homeostasis_scRNAseq_2018.

For manuscripts utilizing custom algorithms or software that are central to the research but not yet described in published literature, software must be made available to editors/reviewers. We strongly encourage code deposition in a community repository (e.g. GitHub). See the Nature Research guidelines for submitting code & software for further information.

Data

Policy information about availability of data

All manuscripts must include a data availability statement. This statement should provide the following information, where applicable:

- Accession codes, unique identifiers, or web links for publicly available datasets
- A list of figures that have associated raw data
- A description of any restrictions on data availability

All original source data are deposited in the Stowers Institute Original Data Repository and available online at http://odr.stowers.org/websimr/publications/libpb-1424

Field-specific reporting

Please select the one below that is the best fit for your research. If you are not sure, read the appropriate sections before making your selection.

☒ Life sciences ☐ Behavioural & social sciences ☐ Ecological, evolutionary & environmental sciences

For a reference copy of the document with all sections, see nature.com/documents/nr-reporting-summary-flat.pdf

Life sciences study design

All studies must disclose on these points even when the disclosure is negative.

Sample size	No statistical analysis was used to calculate sample size
Data exclusions	No data was excluded from the analyses
Replication	*Describe the measures taken to verify the reproducibility of the experimental findings. If all attempts at replication were successful, confirm this OR if there are any findings that were not replicated or cannot be reproduced, note this and describe why.*
Randomization	N/A
Blinding	N/A

Reporting for specific materials, systems and methods

We require information from authors about some types of materials, experimental systems and methods used in many studies. Here, indicate whether each material, system or method listed is relevant to your study. If you are not sure if a list item applies to your research, read the appropriate section before selecting a response.

Materials & experimental systems

n/a	Involved in the study	
☐	☒	Antibodies
☒	☐	Eukaryotic cell lines
☒	☐	Palaeontology
☐	☒	Animals and other organisms
☒	☐	Human research participants
☒	☐	Clinical data

Methods

n/a	Involved in the study	
☒	☐	ChIP-seq
☒	☐	Flow cytometry
☒	☐	MRI-based neuroimaging

Antibodies

Antibodies used	Rabbit Polyclonal Anti-Vangl2 Anaspec AS-55659s (now discontinued); Mouse anti ZO-1 (1:200, Invitrogen #339100); Mouse anti β-II-Spectrin (1:200, BD Transduction, 612562); Rabbit anti-Emx2 (1:250; KO609, Trans Genic, Fukuoka, Japan)
Validation	The antibodies have been previously published (Davey et al. 2016 PLoS Genetics ttps://doi.org/10.1371/journal.pgen.1005934; Kozlovskaja-Gumbriene et al. 2017 eLife 2017;6:e21049 DOI: 10.7554/eLife.21049; Legendre et al. 2008 Journal of Cell Science 2008 121: 3347-3356; doi: 10.1242/jcs.028134; Jiang et al. 2017 eLife 2017;6:e23661 DOI: 10.7554/eLife.23661). These experiments used antibodies from commercial sources, validated according to manufacturer specifications and used in previously published experiments.

Animals and other organisms

Policy information about studies involving animals; ARRIVE guidelines recommended for reporting animal research

Laboratory animals	All experiments were performed per guidelines established by the Stowers Institute IACUC review board. The following mutant fish strains previously described were used: tri^m209 (Stemple et al., 1996), gpc4^fr6 (Topczewski et al., 2001), wnt11 (wnt11f1)^fh224(wnt11r^fh224 in (Banerjee et al., 2011)), wnt11f2^tz216(wnt11/slb in (Heisenberg et al., 1996)), fzd7a^e3 and fzd7b^hu3495 (Quesada-Hernandez et al., 2010), scrib^rw468(a kind gift from C Walsh; (Wada et al., 2005)), wnt5b^t1265 (a gift from D Slusarski; Hammerschmidt et al. 1996).<br>The following transgenic fish lines were used: Tg(cldnb:lynGFP)zf106 (Haas and Gilmour, 2006), Tg(myo6b:[illegible] -Actin-GFP) (Kindt et al., 2012), Tg(cxcr4b:H2A-EGFP) (Kozlovskaja-Gumbriene et al., 2017), Et(krt4:EGFP)sqET4 and Et(krt4:EGFP)sqET20 (Parinov et al., 2004), Tg(cldnB:H2A-mCherry)psi4 (Lush and Piotrowski, 2014a).

Wild animals	N/A
Field-collected samples	N/A
Ethics oversight	This study was conducted in accordance with the Guide of the Care and Use of Laboratory Animals of the National Institute of Health and protocols were approved by the Institutional Animal Care and Use Committees of the Stowers Institute for Medical Research (zebrafish, TP Protocol: # 2017-0176)

Note that full information on the approval of the study protocol must also be provided in the manuscript.
