# Supplementary material for: PCP and Wnt pathway components act in parallel during zebrafish mechanosensory hair cell orientation
Source: Nat Commun. 2019 Sep 5;10:3993. doi: 10.1038/s41467-019-12005-y (PMC6728366; doi:10.1038/s41467-019-12005-y)
Supplement: Supplementary file 10 — Source Data [file 41467_2019_12005_MOESM10_ESM.zip › SourceFile_Uncropped gels.pdf]

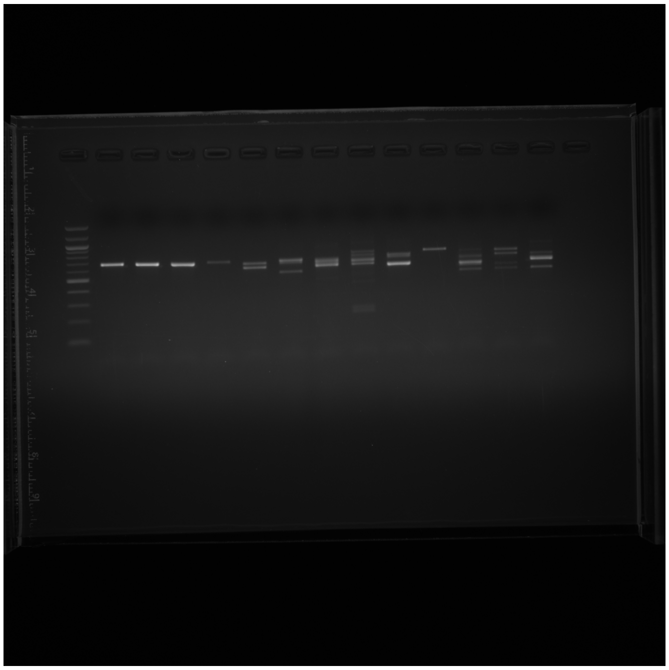

Uncropped gel for Supplementary Figure 2 (top)

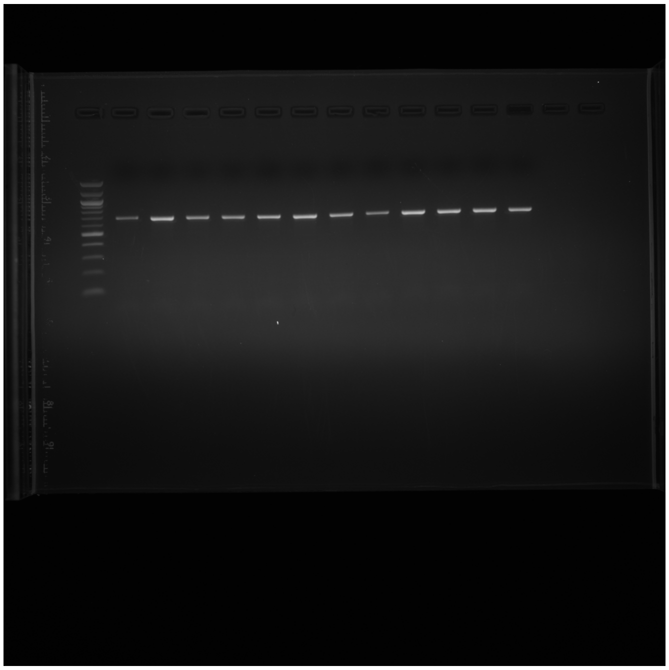

Uncropped gel for Supplementary Figure 2 (bottom)

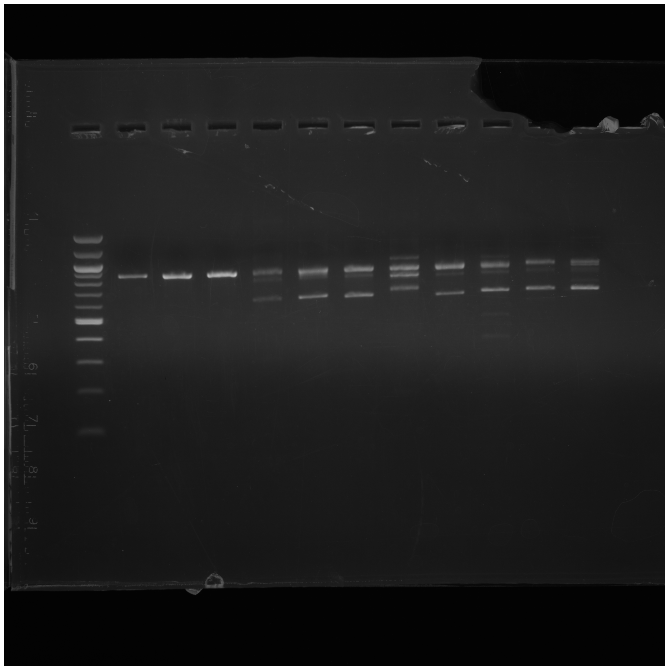

Uncropped gel for Supplementary Figure 5 (top)

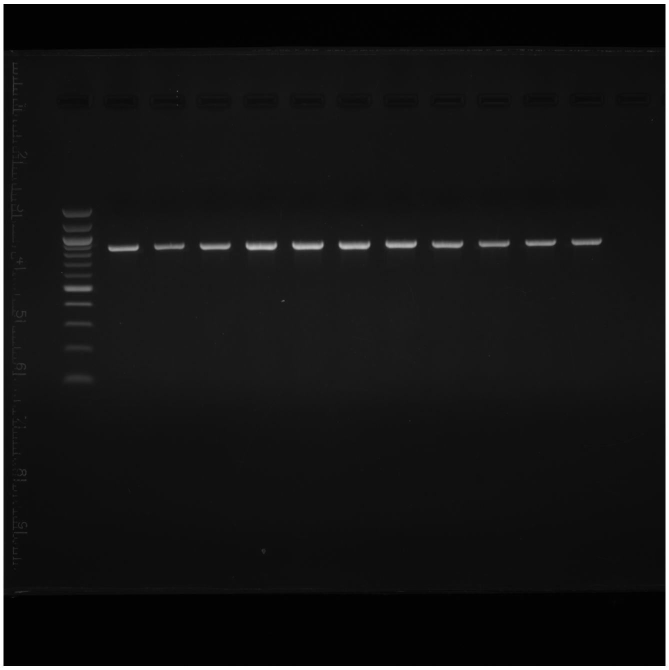

Uncropped gel for Supplementary Figure 5 (bottom)
